# Family-Based Cluster Randomized Controlled Trial Enhancing Physical Activity and Motor Competence in 4–7-Year-Old Children

**DOI:** 10.1371/journal.pone.0141124

**Published:** 2015-10-26

**Authors:** Arto Laukkanen, Arto Juhani Pesola, Risto Heikkinen, Arja Kaarina Sääkslahti, Taija Finni

**Affiliations:** 1 Neuromuscular Research Center, Department of Biology of Physical Activity, University of Jyväskylä, Jyväskylä, Finland; 2 Department of Sport Sciences, University of Jyväskylä, Jyväskylä, Finland; Cardiff University, UNITED KINGDOM

## Abstract

Little is known of how to involve families in physical activity (PA) interventions for children. In this cluster randomized controlled trial, we recruited families with four- to seven-year-old children to participate in a year-long study where parents in the intervention group families (*n* = 46) received tailored counseling to increase children’s PA. Structured PA was not served. Control group families (*n* = 45) did not receive any counseling. PA in all children (*n* = 91; mean age 6.16 ± 1.13 years at the baseline) was measured by accelerometers at the baseline and after three, six, nine and 12 months. Motor competence (MC) (*n* = 89) was measured at the baseline and after six and 12 months by a KTK (KörperkoordinationsTest für Kinder) and throwing and catching a ball (TCB) protocols. The effect of parental counseling on study outcomes was analyzed by a linear mixed-effects model fit by REML and by a Mann-Whitney *U* test in the case of the TCB. As season was hypothesized to affect counseling effect, an interaction of season on the study outcomes was examined. The results show significant decrease of MVPA in the intervention group when compared to the control group (p < .05). The TCB showed a nearly significant improvement at six months in the intervention group compared to the controls (p = .051), but not at 12 months. The intervention group had a steadier development of the KTK when the interaction of season was taken into account. In conclusion, more knowledge of family constructs associating with the effectiveness of counseling is needed for understanding how to enhance PA in children by parents. However, a hypothesis may be put forward that family-based counseling during an inactive season rather than an active season may provide a more lasting effect on the development of KTK in children.

**Trial Registration:**

Controlled-Trials.com ISRCTN28668090

## Introduction

Studies on objectively measured physical activity (PA) indicate that an inactive lifestyle is very common among children and youths [1]. However, an adequate level of PA is vital for normal bone growth [2], developing motor competence [3] and healthy self-esteem [4], and it may play an important role in mental function via psychosocial, physical fitness and general health factors [5]. PA has also been linked with higher cognition [6] and academic achievement [7]. On the other hand, insufficient PA has been shown to be associated with cardiometabolic risk factors [8], as well as decreased psychosocial health in children [9].

PA interventions for children and adolescents with family, school and community involvement have shown small to moderate effects [10–13] or no effects [14] on objectively measured PA levels. While the majority of studies have employed multicomponent intervention methods (i.e. involvement of schools and families simultaneously), there is a lack of knowledge about how best to involve families themselves in PA interventions for children [15,16]. Some evidence of effectiveness has been suggested by interventions with educational and training programs for parents. For instance, “The Healthy Dads, Healthy Kids” [17] educational program with eight face-to-face education sessions for fathers over a period of three months was found to be effective in decreasing the fathers’ weight and increasing the PA of the children. Therefore, more research on effective and feasible family PA intervention strategies would be of great value; parental support of a child’s PA, for example, has consistently been shown to be associated with PA levels in children [18–21].

Because PA behavior has been seen as complex in nature and challenging to change, it is important to research mediative paths supporting an active lifestyle. Development of gross motor competence (MC) has emerged as one major interest in this context. While MC has multifaceted associations with PA [3,22], it also predicts the level of PA [23,24] and fitness [25], and it is associated with perceived sports competence, which mediates the level of PA later in life [26]. It has also been shown that acquired MC itself may act as a mediator for increased PA [27]. On the other hand, low MC is hypothesized to be one factor predisposing a physically inactive lifestyle and accumulation of health risk factors [28]. Although behavioral theories (e.g. social cognitive theory [29]) and some evidence consider the influence of the home environment to be important on the development of MC in children [30–32], little is known about whether not only habitual PA patterns, but also the development of MC could be influenced by family-based intervention. At best, a home or parental component has comprised only a minor area of study in efforts to enhance PA and MC in children [33,34], making it difficult to interpret the effect of family on the outcomes.

The present cluster randomized controlled trial addressed this gap by testing 1) whether family-based tailored counseling aimed at increasing PA in children is an effective way to enhance objectively measured PA in children. The secondary goal was to examine 2) whether family-based counseling is effective in contributing to the development of MC in children. Additionally, it has been shown that seasonal variation may significantly affect PA behavior and fitness in children [35,36]. Because this study was conducted in a northern country with great seasonal variation and possible effects (e.g. frequency of outdoor PA), we also examined 3) whether seasonal variation played a role in the effects of counseling on changes in PA and MC in children. The advantages, challenges and limitations of family-based PA counseling are discussed on the basis of the findings of this unique study and previous literature on PA interventions with children. Also, an interaction between season and the study effect on KTK performance is proposed as one means of strengthening the effect of family-based PA counseling on MC development in children.

## Materials and Methods

This study was conducted as part of a year-long randomized controlled trial “A family-based tailored counseling to increase non-exercise physical activity in adults with a sedentary job and physical activity in their young children” (InPact, ISRCTN28668090) [37]. Overall, the InPact study was aimed at increasing non-exercise PA in adults and PA in their young children via individually tailored PA counseling. In this paper, the counseling process and main outcomes regarding the children are reported. The authors confirm that all ongoing and related trials for this study are registered. A delay in the registration of the trial was due to time constraints in the study implementation. Ethical approval for the project was received from the Ethics Committee of the Central Finland Health Care District on March 25, 2011 (Dnro 6U/2011) and we obtained written informed consent from all of the parents for their own and their children’s involvement in the study. Reporting of the methods and findings of this trial was guided by a checklist of the CONSORT 2010 Statement for reporting randomized trials [38].

### Cluster randomization and recruitment

**Fig 1:**
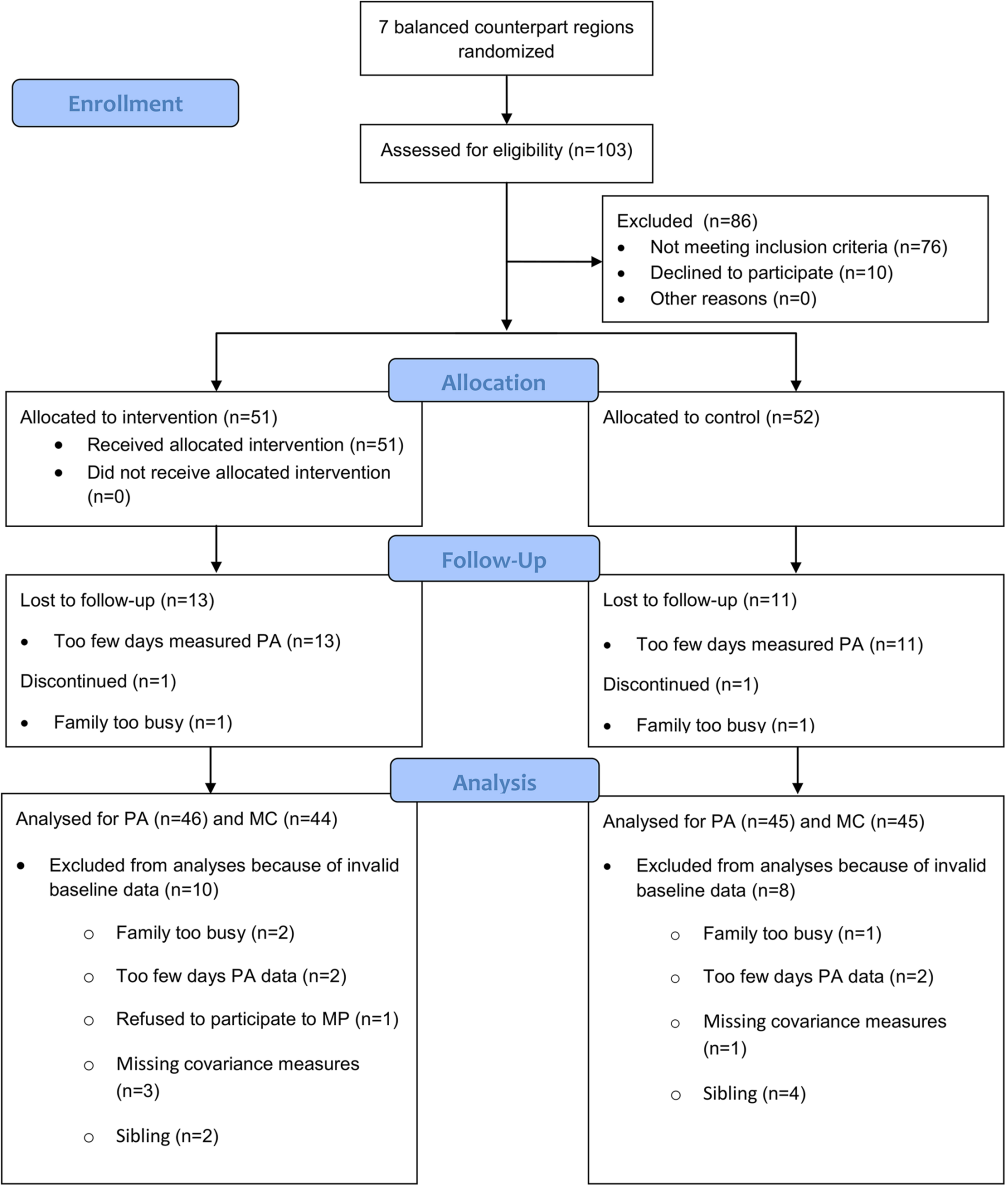
Flow chart of the study. PA, physical activity; MC, motor competence.

The study was performed in a city of Central Finland with approximately 133,000 inhabitants living in a relatively small city center and topographically and socioeconomically varied suburbs. Balanced regions in the city (henceforth referred to as “clusters”) were identified in terms of population, daycare centers and school facilities, socioeconomic characteristics (education) and outdoor PA possibilities. Seven balanced counterpart clusters were formed (from one to four daycare centers or schools in each cluster) and randomization into either intervention or control clusters was done by researchers (AL, TF) for each of these counterparts. As a result, there were seven intervention clusters and seven control clusters. Recruitment of families for the intervention group was then performed from the intervention clusters and families for the control group from the control clusters. The allocation ratio was around 10%, with 1055 recruitment letters sent to parents via children attending 21 daycare centers and eight primary schools. Altogether 101 children were allocated to the study. The researchers (AL, AP, TF) performed randomization, enrolled participants, and assigned participants to the study. The flow of participants through the cluster randomized controlled trial is illustrated in Fig 1. Children attending daycare less than 10 days a month, children with a developmental disorder or other disorders delaying motor development, children whose parents sat less than 50% of their work time or had a chronic disease, and children with a pregnant parent were excluded. At least one parent and a child were required for the family to be included in the study. The recruitment of participants was performed between the 1st of April, 2011 and the 30th of April, 2012. The baseline measurements took place between the 2nd of May, 2011 and the 2nd of May, 2012 in a balanced manner for the intervention and control group families. All parents were given the possibility to receive PA counseling: intervention families after the baseline measurements and control families after the final measurements.

### Tailored counseling

Tailored counseling to support parents in changing behavior to increase PA in their children was based on social cognitive theory (SCT) [29] and the theory of planned behavior (TPB) [39]. The TPB was added to the study design after commencement of the trial in order to complement the tailored counseling process. The behavior change techniques used in this study were based on nine items conducted in one or several parts of the counseling process: 1) a lecture, 2) individual face-to-face counseling and goal setting, and 3) counseling by phone (Table 1). The lecture and individual discussions were led by researchers (AL, AP, TF) who had all undergone an orientation in best practices in behavior change counseling before the study. The phone counseling sessions were conducted by two researchers (AL, AP). In the lecture, parents were instructed that outside the daycare or school context, one hour of moderate-to-vigorous PA (MVPA) during weekdays and two hours of MVPA during the weekend days was the target level of PA (Table 1: item 1). This general target was justified by the gap between national PA guidelines and preliminary research findings about the current level of PA in children [40] and by the assumed consequences of (not) achieving the recommended PA. Specifically, the close relationship between PA and health, the development of MC, and school readiness were explained to parents (Table 1: item 2). Scientific-based, concrete strategies for enhancing PA in children were discussed. The key message was to enable PA in a way natural for children (e.g. running around and climbing, not restricting them unnecessarily) and also to offer possibilities for PA in non-constructed environments–such as heaths, forests and hills–as time spent outdoors associates with PA and PA in natural environments may contribute the development of balance and motor coordination [41,42]. Seasonal variation, and especially the decline of PA in late autumn and winter, was emphasized as a key challenge for PA in children (Table 1: item 1). The role of parents as an important model for their children’s PA behavior in everyday life, and not only regarding exercise habits, was emphasized. Parents were encouraged in PA-friendly role modeling (Table 1: item 3). Typical restrictions put against PA in children’s everyday life were discussed by parents and researchers during the counseling session (Table 1: item 5).

**Table 1:** Description of the techniques used in the family-based PA counseling.

Technique item (theoretical framework)	Counseling	Description	Example of implementation
1 Provide instruction (SCT)	Lecture, face-to-face, phone counseling	Providing scientific-based ways to increase PA in children	“Outdoor PA, PA with peers, PA with parents, active ways of commuting”
2 Provide information on consequences (SCT, TPB)	Lecture	Information about how physical activity enhances health, development of gross and fine motor coordination, and therefore academic readiness	“PA is associated with lower cardiometabolic risk factors in children, and lack of gross motor coordination may hamper development of fine motor coordination.”
3 Prompting identification as a role model (SCT)	Lecture	Information of concrete situations where parents act as physically active role models for their children	“Consider if you could choose stairs instead of a lift and walking instead of taking a car.”
4 Provide general encouragement (SCT)	Lecture, face-to-face, phone counseling	Justifying concrete benefits from the intended behavior change	“Adequate PA during the day helps children to go to sleep.”
5 Provide information about others approval (TPB).	Lecture	Information about other parents’ and authorities’ opinions/rules about restricting PA that is natural for children	Discussion of typical restrictions with other parents (e.g. restricting children from running up stairs, playing ball outdoors in rainy weather and climbing on trees).
6 Prompting intention formation (SCT, TPB)	Face-to-face	Encouragement for enabling behavior change	“Consider if prohibiting children from jumping indoors would be unnecessary.”
7 Progressive goal setting (SCT)	Face-to-face, phone counseling	Encouragement to set target frequency for goal implementation, prompting for considering progressive increase of the target frequency	“I aim to provide my children with weekly opportunities for outdoor play during leisure time.”
8 Prompting barrier identification (SCT)	Phone counseling	Prompting parents to identify barriers of PA in children and implementing the goals set in the counseling session	“What are the reasons your child was not able to play outdoors on the weekend?”
9 Self-evaluation	Phone counseling	Parents were asked to self-evaluate the implementation of goals that were set	“On a scale of 1–5, how well did you do in achieving the set goal?”

PA = physical activity; SCT = Social Cognitive Theory; TPB = Theory of Planned Behavior; Face-to-face = face-to-face discussion between parent and counselor.

Following a fidelity checklist of the individual face-to-face discussion, parents were first asked to describe the PA habits of the family during leisure time, and then encouraged to consider and set small, gradual goals for increasing the children’s PA to reach the target level. Physical activities common to the entire family were also encouraged (Table 1: items 4 and 6). The goals that parents set were rated on a scale of 1 to 4, depending on the frequency of intended implementation (1 = randomly, 2 = once or twice a week, 3 = three to four times a week, 4 = daily) (Table 1: item 7). The goals set by the parent him/herself were written as an agreement that was signed by the parent and the researcher.

To promote compliance with implementation of the goals, phone discussions were held at two and five months after the counseling and goal setting. During the phone calls, compliance with the goals set, possible modifications to the goals, and perceived barriers to implementation of the goals were discussed (Table 1: item 8). Additionally, the parents were asked to self-evaluate the implementation of goals by answering the question “Did you do your best to achieve the goal?” on a scale of 1 to 5 (1 = not at all, 2 = a little, 3 = moderately, 4 = relatively well, 5 = fully) (Table 1: item 9). Implementation was supported by monthly e-mails which contained seasonal tips and illustrative videos about how to increase PA and develop MC in their children. Feedback about the progress of a child’s MC in comparison to age-related peers was given to parents shortly after the six-month measurements. The feedback form also included practical advice for improving MC (e.g. moving on varied terrain enhances the development of balance and coordination). The last six months of the study were the same for the intervention and control groups, including only nine- and 12-month assessments but no other contact with the researchers.

### Assessment of PA, MC and anthropometrics

PA was measured using triaxial X6-1a accelerometers with a dynamic range of ±6 g (Gulf Coast Data Concepts Inc., Waveland, MS, USA) at the baseline and three, six, nine and 12 months for six consecutive days at a time. Recordings longer than 7 hours (420 minutes) on at least 3 days (2 weekdays and 1 weekend day) [43] were accepted for analysis. Proportional values of time spent at different PA intensities of sedentary, light and MVPA [44] were calculated in relation to the total measurement time and by weighting weekdays by 5/7 and weekend days by 2/7. Three control children were lost for PA follow-up at three months, five intervention and three control children at six months, six intervention and five control children at nine months, and two intervention and one control children at 12 months because of too few days measured PA (Fig 1). Missing PA values were imputed by using a predictive model on the condition that the subject had successfully performed the baseline and at least one other measurement point. Variation in school timetable, teacher support for PA, etc. may cause bias on the study effects; therefore, PA during school time, imputed when needed, was used as a covariate when explaining the change in MC due to counseling. The imputed PA was not used as a dependent variable itself, but only as a predictive covariate.

MC was measured at the baseline, six and 12 months, using the Körperkoordinationstest für Kinder (KTK), standardized in Germany in 1974, which has been shown to be highly reliable [45]. The KTK consists of four different test items: walking backwards (WB), hopping for height (HH), jumping sideways (JS) and moving sideways (MS). The means of the raw scores of the four KTK items at each measurement point were calculated and used as a secondary outcome measure. Additionally, a throwing and catching a ball test (TCB) from an APM inventory was used for measuring ball-handling skills. The APM inventory has been validated in 1800 Finnish children of under eight years of age, and it has been shown to be highly reliable (test-retest *r* = 0.86–0.94) [46]. In this study, the TCB for preschool children (aged four to six) utilized a soft ball (circumference 65.4 cm; weight 228 g), which was thrown underarm 10 times towards a target (10-cm wide piece of distinguishable tape) at a height of 1.3 m on the wall from a distance of two meters and caught after a bounce on the floor. The TCB was modified for primary schoolers (aged seven) so that it was performed in two separate parts with a higher degree of difficulty. In the first part, the ball was thrown 10 times from a distance of three meters and caught after a bounce on the floor. Additionally, hits on the wall above the marked two-meter upper limit were counted as fails. In the second part, the ball was thrown 10 times from a distance of three meters and caught without a bounce on the floor. No upper limit was marked on the wall for the second part. The number of catches was summed for preschoolers, and for primary schoolers the average number of catches in the two parts was calculated. Performing the KTK and TCB took approximately 20–30 minutes per child, and testing sessions took place either in the laboratory, in daycare center, or at school in groups of one to three children. The testing conditions were as similar as possible in every case regarding distractions, floor material and space needed for the measurements.

Height and body weight were measured in the laboratory at six and 12 months, and BMI (kg/m^2^) was calculated. The BMI did not significantly change over time and thus the six-month BMI was used in statistical analyses. Based on international cutoff points [47], approximately 11% of children in this study were overweight.

### Statistical analysis

Differences between groups (intervention and control) and gender in terms of background characteristics were tested by independent samples T- and chi-square (*X* ^*2*^ *)* tests. The effect of counseling on PA and the KTK was analyzed with a linear mixed-effects model fit by REML using statistical programming language R (R 3.0.1, NLME package, the R foundation for Statistical Computing). An autoregressive covariance model (AR1) was also used in the analyses considering changes in the KTK.

Analysis of the counseling effect was initially based on a three-level hierarchy where children (*n* = 97) were nested within families (*n* = 91) and families were nested within randomized clusters (*n* = 14). The children, families and clustered samples were considered in the models as random grouping effects. However, the models were inestimable with the family-level hierarchy because of the great number of families in comparison to the total number of children. Therefore, in five cases where more than one child per family was participating to this study, only one child from the family was randomly included to the final analyses. Consequently, the final counseling effect analysis based on a two-level hierarchy where children (*n* = 91) were nested within randomized clusters.

The Group × Time interaction formed a base model for examining the effects of counseling on the proportional change of time spent in different PA intensities and the KTK between the baseline and the 12-month follow-up. Based on this interaction, the mean change from the baseline to six months and the baseline to 12 months, and the mean difference between groups in these time intervals, were calculated. In the second phase, the interaction of gender was added to the base model and the three-way interaction of Group × Time × Gender was tested with the Likelihood ratio test. The models with and without the three-way interaction term were compared. The same procedure was applied for the three-way interaction of Group × Time × Season in order to examine the influence of seasonal variation on the study effects. Subjects were divided into three groups based on the season when they were tested at the baseline: *spring* (*n* = 30) (March, April, May and June), *autumn* (*n* = 42) (August, September, October and November) and *winter* (*n* = 22) (December, January and February). The influence of seasonal variation was illustrated by plotting the proportion of time spent in MVPA at the baseline, three, six, nine and 12 months, and the mean of KTK and TCB at the baseline, six and 12 months among intervention and control groups, starting in spring, autumn and winter. Following the intention-to-treat principle, all subjects with acceptable baseline data from outcome measurements and covariances were included in the analyses of study effect. From the total of 101 children, the effect of counseling on PA (Group × Time; Group × Time × Gender; Group × Time × Season) was analyzed with 48 intervention and 49 control children with the intended treatment, and the study effect on the KTK (Group × Time; Group × Time × Gender; Group × Time × Season) with 46 intervention and 49 control children.

All mixed models were adjusted for theory-based confounding variables (in order of statistical importance, with PA as dependent variable: average monthly temperature, participation in extracurricular PA, gender, age and season at baseline measurement; with KTK as dependent variable: age, BMI, proportion of time spent in MVPA during school time, participation in extracurricular PA, and testing environment). Average temperatures were retrieved from climate statistics by the Finnish Meteorological Institute. Sedentary time was LOGIT-transformed, while light PA and MVPA were LOG-transformed due to skewed distributions. Furthermore, distribution of the TCB was not normal at the baseline because of several zero-point performances (*n* = 13) in the youngest participants. Therefore, a related samples Wilcoxon signed rank test (*W*) was used to examine the development of TCB by time in general. Non-parametric Mann-Whitney *U* test was used for testing differences between the groups in changes of the TCB: first, in all children, and secondly, in girls and boys separately A logistic regression analysis was performed for revealing possible systematic explanations (e.g. parents’ education level) for dropping out of the study. The level of significance was set to p < .05 in all analyses.

## Results

### Participant characteristics and measurement flow

The proportion of overweight children included in this study (11%) was within the national average for five-year-olds (9.8–17.7%) [48]. Intervention group accumulated significantly less sedentary time (*t* = 2.23, p = .028) and more MVPA (*t* = 2.52, p = .013) at baseline (Table 2). Boys cumulated significantly less sedentary time (*t* = 2.78, p = .007) and more light PA (*t* = 3.64, p < .001) and MVPA (*t* = 2.02, p = .047) compared to girls at the baseline, but MC was similar between genders. Parents in the intervention group were significantly older than in the control group (*t* = 3.37, p = .001). When compared to the mean of the whole recruitment region, parents in this study were more highly educated (i.e. they were more likely to have a university or polytechnic degree (71% / 35%) and less often single parents (4% / 27%). There were no other significant differences between gender or the intervention and control groups in terms of background characteristics or baseline assessments.

**Table 2:** Background characteristics of the children and parents for analysis.

Characteristics	Intervention	Control
Children (*n*)	46	45
Girls (n)	25	24
Age (years)	6.07 ± 1.12 (3.65)	6.20 ± 1.13 (3.60)
Height (cm)	121.10 ± 7.53 (34.20)	120.21 ± 7.83 (29.70)
Weight (kg)	23.31 ± 3.46 (15.0)	22.66 ± 4.06 (16.6)
BMI	15.84 ± 1.18 (5.90)	15.58 ± 1.50 (8.89)
Season enrolled in the study		
Spring (n)	18	13
Autumn (n)	17	22
Winter (n)	11	10
Physical activity (n)	46	45
Sedentary (%)	87.51 ± 4.05 (17.95)*##	89.25 ±3.33 (14.93)
Light (%)	5.44 ± 2.44 (8.12)###	5.08 ± 1.40 (6.84)
MVPA (%)	7.11 ± 2.94 (13.31)*#	5.73 ± 2.21 (10.11)
Motor competence (n)	44	45
KTK	30.09 ± 12.80 (48.0)	31.02 ± 11.50 (41.75)
TCB	4.47 ± 3.04 (10)	4.72 ± 2.91 (10)
Parents involved in the study (n)	64	58
Age	36.34 ± 4.88 (25)**	39.48 ± 5.40 (22)
Females (n)	40	30
Higher-level education (%)	67.04 (%)	67.78 (%)
Household income ≥ 60 000€ (%)	62.79 (%)	58.14 (%)
Single parent (%)	2.22 (%)	4.65 (%)

Data are presented as mean ± SD and range (in parentheses) from the baseline measurements, except height, weight and BMI (kg/m^2^) for children, which are presented from the midline measurements.

Season, season when enrolled in this study; KTK, mean value of all four items of the KörperkoordinationsTest fur Kindern; TCB, mean score of throwing and catching a ball.

Significant difference between intervention and control groups, p < .05 (*), p < .01 (**) and between genders, p < .05 (#), p < .01 (##), p < .001 (###).

On average, PA was measured for 5.04 days (11.79 ± 0.93 h/d), 5.17 days (11.74 ± 0.93 h/d), 5.22 days (11.84 ± 0.98 h/d), 5.15 days (11.59 ± 0.85 h/d) and 5.27 days (11.68 ± 0.90 h/d) at the baseline, three, six, nine and 12 months, respectively. A total of 3 intervention and 4 control children discontinued the study after enrollment because of a busy life situation in the family or parents participating in another study (Fig 1). Four children (three intervention and one control) were excluded from the analysis of study effects on the KTK because of one or more missing covariance measurements. Six children (two intervention and four control) were dropped out from all intervention effect analysis because of a sibling(s) taking part to the study. Study dropouts did not statistically differ from other subjects involved in the study.

### Effect of the study on PA

Group × Time interaction indicated a significant decline of MVPA (*D* = 10.45, *df* = 4, p = .033) in the intervention group when compared to the control group (Table 3). Group × Time × Gender interaction indicated no significant gender differences in the treatment effect on the proportion of time spent at different PA intensities.

**Table 3:** Change in physical activity and motor competence for intervention and control groups at 6 and 12 months.

			Mean change (95% Confidence Interval)		Mean difference between groups (95% Confidence Interval)	P-value			
Outcome		Period of change in months	Intervention	Control	Intervention–Control	Time	Group × Time	Group × Time × Gender	Group × Time × Season
Physical activity									
	Sedentary (%)	0–6	0.04 (-0.07 to 0.16)	-0.07 (-0.18 to 0.04)	0.11 (-0.03 to 0.26)				
		0–12	0.02 (-0.11 to 0.15)	-0.10 (-0.22 to 0.03)	0.11 (-0.02 to 0.25)	.506	.106	.642	.171
	Light (%)	0–6	0.02 (-0.07 to 0.11)	0.04 (-0.05 to 0.13)	-0.02 (-0.14 to 0.10)				
		0–12	0.04 (-0.06 to 0.15)	0.06 (-0.04 to 0.17)	-0.02 (-0.13 to 0.09)	.775	.285	.511	.200
	MVPA (%)	0–6	-0.11 (-0.24 to 0.02)	0.08 (-0.05 to 0.21)	-0.19 (-0.35 to 0.02)				
		0–12	-0.08 (-0.24 to 0.08)	0.08 (-0.08 to 0.24)	-0.16 (-0.32 to 0.001)	.172	**.033**	.507	.212
Gross motor coordination									
	KTK	0–6	18.80 (13.74 to 23.86)***	17.39 (12.22 to 22.56)***	1.41 (-5.89 to 8.71)				
		0–12	35.28 (29.6 to 41.0)***	36.76 (30.97 to 42.54)***	-1.47 (-9.52 to 6.58)	**< .001**	.737	.930	**.008**

*** Significant change within group, p < .001.

### Effect of the study on MC

**Fig 2:**
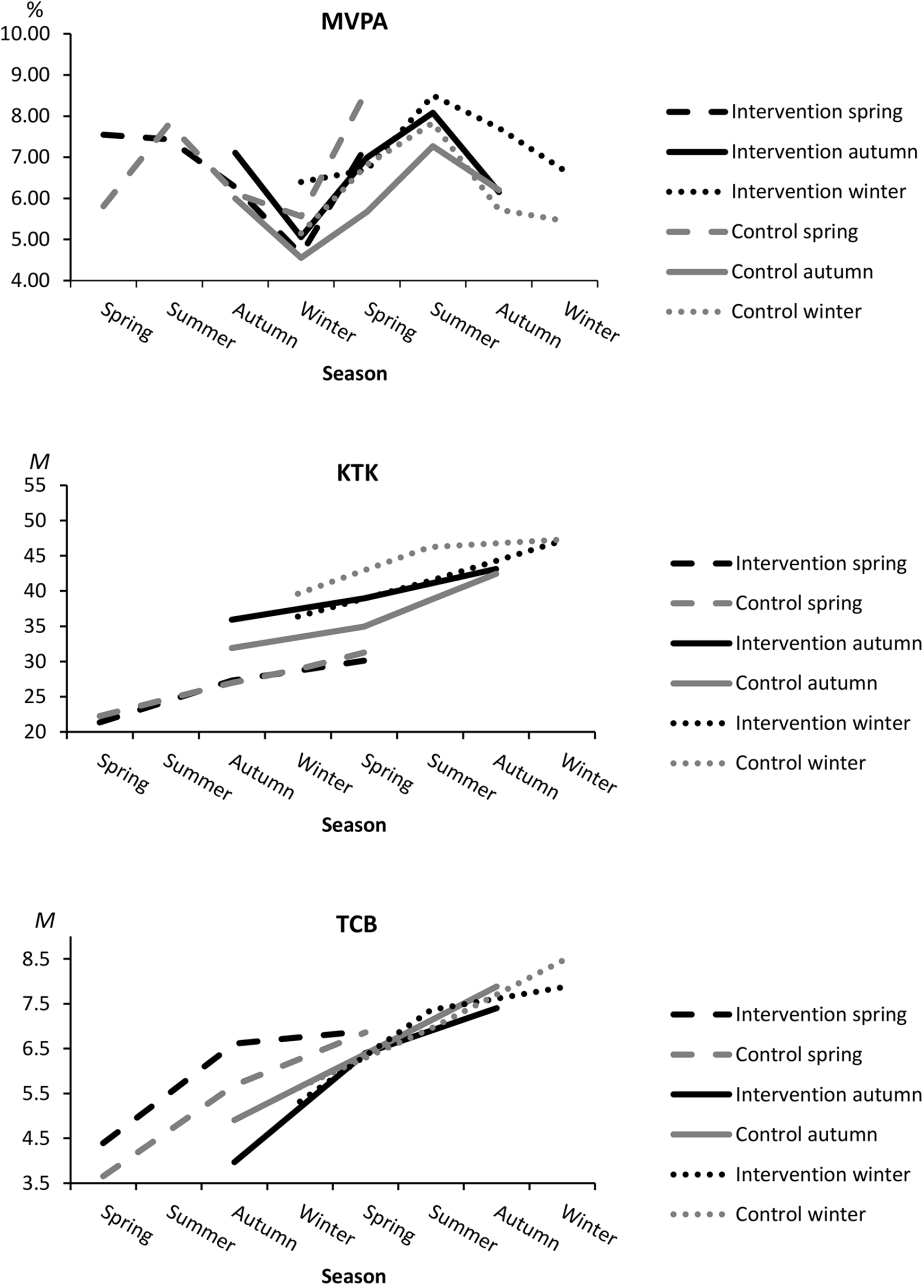
Seasonal variation in intervention and control groups starting in spring, autumn and winter in relation to proportional time spent at MVPA, and the development of the mean of the KTK and TCB. Season is plotted on the x-axis and the response variable on the y-axis. MVPA, moderate to vigorous physical activity; KTK, Körperkoordinationstest für Kinder; TCB, throwing and catching a ball.

The mean score of KTK (*F* = 154.5, p < .001) and TCB (*W* = 7.46, p < .001) increased significantly with time (Table 3, Fig 2). Group × Time interaction showed no study effect for the development of the KTK. There were no significant differences between genders in the study effect for the development of the KTK. The TCB indicated a slightly greater, although not quite significant, improvement among intervention group (increase of 2.25 ± 2.34 points) compared to the control group (increase of 1.34 ± 2.40 points) between the baseline and six months (*U* = 753.5, p = .051). The change of the TCB did not differ between groups from the baseline to 12 months (*U* = 987.5, p = .984). When genders were analyzed separately, there were no significant differences between groups in the development of the TCB (data not shown).

### Influence of the season on study effects

Group × Time × Season interaction in the KTK (*D* = 23.97, *df* = 10, p = .009) indicated a significant study effect on the KTK when taking the influence of season into account (Table 3). More specifically, intervention and control groups who started the study during winter differed in the progress of the KTK during the transition from an active to inactive season in the latter half of the follow-up (from six to 12 months, difference of 11 percent points and seven raw points) (Fig 2). Season had no significant interaction effect on the changes of PA between groups.

### Study evaluation

Every parent (*n* = 69) in the intervention group received a lecture (~ 30 minutes) and face-to-face counseling with goal setting (~ 30–60 minutes). Of those, 64 (93%) and 51 (74%) were reached for phone discussions at 2 and 5 months, respectively (Fig 1). During the counseling session, parents set an average of 3.5 goals intended to increase their child’s PA. The most common goals were: PA with the family (28%), PA outdoors (25%), PA in the backyard or in the neighboring area (22%), PA with peers (18%) and PA indoors (6%). The parents who were reached once or twice for phone counseling perceived the most common weekday barriers to goal implementation to be weather (38%), hurry and needing to do other tasks (30%), and either their own or the child’s tiredness (17%). On weekends, hurry and needing to do other tasks (35%), weather (21%) and tiredness (10%) were the most common barriers. Both mothers and fathers in the intervention group rated individual discussion as the most important study tool used in this project (~ 32% of the parents), followed by feedback from measurement results (~ 19%), the lecture (~ 18%), phone discussions (~ 3%), printed material (~ 4%), emails (~ 4%) and project web pages (0%).

## Discussion

The current year-long RCT showed that a single counseling session given to parents and accompanied by reinforcing phone calls and e-mail contacts could not increase but rather showed decrease in objectively measured MVPA in children aged 4–7 years. On the other hand, although ways to influence development of children’s MC was not primarily targeted in the present family based PA counseling, a greater development of KTK was found in the intervention group when the interaction of season was taken into account. Furthermore, a positive change in ball-handling skills, even though just below the level of significance, was observed in the intervention group (TCB) during the reinforced counseling period (0–6 months). Results suggest that family-based PA counseling may have distinct influences on PA behavior and on the development of MC.

The results of the present study parallel previous interventions employing parents as promoters for PA and MC in their own children. In the study of Hamilton et al. [49], children at risk of developmental delay significantly outperformed their control peers in ball-handling skills after an investigator-led and mother-assisted eight-week motor skill program. Similarly, Cliff et al. [34] recruited obese children to participate in structured PA sessions led by qualified PE teachers over ten weeks. Families were educated to enhance social support for PA, to monitor behavior, to identify barriers for PA, and to set goals enhancing PA in their obese children. As a result, motor skills improved significantly in subjects compared to control peers, but objectively measured PA remained unchanged between groups. Therefore, family involvement is an important component of treatment when aiming to enhance gross motor development, while improved MC may act as a mediator for increased PA [27]. However, it is crucial to further also research direct strategies for affecting PA, as interventions aimed at increasing PA in children have generally only produced modest results [14]. We clearly need more knowledge about how to effictively involve families, for example, in enhancing PA in children.

Although the present study did not show significant effect on TCB in children, there is a need to find ways to support the development of object control skills in children. Object control skills (i.e. ball-handling skills) have been stated to be more difficult to change than locomotor skills [50] and, most importantly, acquired ball-handling skills have been shown to predict PA and fitness later in life, especially in girls [23,25]. However, as previous school-based intervention studies have shown good sustainability of acquired MC in children [51,52], the nearly significant development of object control skills in the present family-based intervention group was attenuated after a reinforced counseling period in the present study. It can be speculated that family-based PA counseling itself does not guarantee a sustained development of ball skills in a school context, for example, because girls generally have less ballgame-oriented lessons at school [53]. Therefore, a home setting should be seen as a potential reinforcer for the development of ball skills. As the MC was only indirectly targeted, a family based counseling targeting ways to support the development of MC, e.g. ball handling skills, would more likely influence the proficiency of these skills in children. It should be further investigated whether increase of the overall level of PA would mediate the improvement of MC in children as the level of PA remained unaffected in the present study and does not therefore provide evidence for or against the question. Educational, curricular and other environmental contexts should ideally be shifted at the same time to strengthen the sustainability of skill development.To our knowledge, this is the first study to suggest a significant interaction between seasonality and PA counseling effects on the development of MC in children of the intervention group. Interestingly, the family based PA counseling had a simultaneous negative influence on PA behavior. The explanation for this can be found from the multifaceted relationship between PA and MC. Because the development of object control skills requires training of these specific skill domains and they are not simply the result of accumulated PA [31,54], it is possible that accelerometer-based PA monitoring is not able to detect this kind of PA accurately enough. Regarding the development of KTK, accelerometer-derived sedentary- to light-intensity PA, along with MVPA, may be associated with physical activities typically seen as developing components of MC in children [55], and thus it may be difficult to capture some developmentally appropriate PA via typical objective PA measurements. Additionally, aside from the proportion of time spent in MVPA, brief but high impact peaks may play a role in the development of MC [22]. Clearly, a more comprehensive interpretation of accelerometer-derived PA from the point of view of motor development warrants future study. Also, self-reports or parent-reports would have been useful supplementary tools for assessing, for instance physically active time spent outdoors and in natural environments, as children may engage in activities that help their MC development but are not reflected in the objective measurements.

For counseling planning, some important issues emerged from the present study. Individual face-to-face counseling was considered as the most useful study tool among the parents in the intervention group. This finding endorses the value of parent-authority interaction and justifies the use of MC targeted counseling for parents, for instance, as a part of maternity and child welfare clinic visits. Additionally, parents rated the initial lecture and feedback on the measurement results among the most important study tools. Parents would probably gain even more from instant feedback about children’s MC and practical advice on how to increase the development of MC in their children. On the other hand, a significant decrease in MVPA in the intervention group compared to the control group was unexpected, although there are some parallel findings [11]. Perhaps the increased time spent with family was compensated for with decreased time spent with peers, which may have led to a decrease in overall physically active play. More specifically, as the importance of PA in diversified outdoor environments was highlighted in the counseling process, it may be that time spent, for instance, in forests instead of parks may partly explain the compensation of accelerometry-derived MVPA by PA of lighter intensity. Time spent in diversified environments might also associate with the significant intervention effect on the KTK performance. On the other hand, it may be that the advance knowledge of being part of a study where PA counseling is given may have induced an unwanted treatment effect already at the baseline PA assessments potentially explaining the significantly higher baseline level of MVPA and lower baseline level of sedentary time in children of the intervention group compared to the control group. However, these explanations remain speculative.

The strengths of this study were 1) a unique family-based counseling approach, 2) frequent and objective measurement of the main study outcomes, and 3) following of the intention-to-treat principle by keeping dropouts in the analyses of study effects, decreasing potential bias caused by selectiveness of drop-outs.

There are some limitations to take into consideration in this study. The KTK measurement protocol is designed for children aged 5–15, but children under 5 years of age at the baseline (*n* = 9) were also included in this study. Because the developmental rate of the KTK was statistically consistent between children under and over 5 years old, the inclusion of children under 5 was considered justified. Secondly, different study protocols were used to measure the TCB in younger and older children, and the distribution of the TCB was not normal at the baseline. Therefore, non-parametric tests were used to examine differences between groups in terms of changes in the TCB. Additionally, the clustered samples were not taken into account in the non-parametric analysis which has to be understood as a limitation of the study. However, the change in the TCB was generally greater in the intervention group compared to the control group between the baseline and the end of the reinforced counseling period. This favors a real study effect, although just below the level of significance, in change of the TCB. Lastly, the families included in this study were highly educated and, therefore, the results cannot be generalized to less educated families.

In conclusion, family-based counseling was found to decrease objectively measured MVPA but to increase motor coordination as measured using KTK in children. The findings indicate that there is a lack of knowledge how children’s PA can be enhanced by parents. However, the present study suggests that initiation of family-based PA counseling during the inactive season may induce a more sustainable effect on the development of KTK performance.

## Supporting Information

S1 CONSORT Checklist CONSORT checklist. (DOCX)

S1 Protocol Clinical trial protocol. (DOCX)
