# Correction: Family-Based Cluster Randomized Controlled Trial Enhancing Physical Activity and Motor Competence in 4-7-Year-Old Children

**DOI:** 10.1371/journal.pone.0143987

**Published:** 2015-11-23

**Authors:** Arto Laukkanen, Arto J. Pesola, Risto Heikkinen, Arja Kaarina Sääkslahti, Taija Finni

The second author’s name is incorrectly spelled. The correct name is: Arto J. Pesola.

## References

[1] Laukkanen A, Pesola AJ, Heikkinen R, Sääkslahti AK, Finni T (2015) Family-Based Cluster Randomized Controlled Trial Enhancing Physical Activity and Motor Competence in 4–7-Year-Old Children. PLoS ONE 10(10): e0141124 doi:10.1371/journal.pone.0141124 26502183 10.1371/journal.pone.0141124 PMC4621056

