# Supplementary material for: Family-Based Cluster Randomized Controlled Trial Enhancing Physical Activity and Motor Competence in 4–7-Year-Old Children
Source: PLoS One. 2015 Oct 26;10(10):e0141124. doi: 10.1371/journal.pone.0141124 (PMC4621056; doi:10.1371/journal.pone.0141124)
Supplement: S1 Protocol — (DOCX) [file pone.0141124.s002.docx]

**Minimizing inactivity periods and increasing non-exercise physical activity in parents and their young children**

InPact study plan for ethics committee February 2011

Our physical activity level is determined by genetic (Pellegrini & Bjorklund 1998) and environmental factors (Gallahue & Ozmun 2006, Malina ym. 2004). Especially, learning physical activity habits from parental model has been shown to be influential (Sääkslahti 2005). Thus parents are in key position to influence children’s physical activity habits based on social cognitive theory of learning (Bandura 2002). On the other hand it has been reported that adults with young children are least active in Finland (Rovio et al. 2009). Thus, parents and their young children are the primary target of the present intervention study focusing especially on non-exercise physical activity.

By definition, physical activity is bodily movement that is produced by the contractile activity of skeletal muscle that substantially increases energy expenditure (ACSM). Heart rate (HR) elevation has been considered the prime response to physical activity and it is easily assessed even long periods of time. Heart rate recordings are typically used to evaluate cardiovascular response. However, HR is affected also by other factors than physical activity (Tulppo et al. 2003). Indeed, it is intriguing to think that HR and oxygen uptake are never zero, although person is not doing any physical activity (Fig. 1). On the other hand, muscle activation is prerequisite for physical activity but its role in quantifying physical activity level has been neglected. This study uses electromyography (EMG) to accurately measure muscle activity – and lack of activity – in ordinary people (Finni et al. 2007, Finni et al. 2010ab). Hypotheses on inactivity physiology paradigm that sitting is harmful for health even if person is considered to be physically active (Hamilton et al. 2007) can be readily examined with EMG measurements that reveal periods of sitting and quiet standing (Fig. 1). A report indicates that breaks in sedentary time independently of moderate-to-vigorous physical activity is beneficially associated with health indices such as BMI and triglycerides (Healy et al. 2008). Nationally this is very relevant issue, since 46% of women and 51% of men sit daily at least 6 hours (Sjöström et al. 2006).

Fig.1. Simultaneous recordings of HR and EMG from thigh muscles when standing quietly and during stair climbing and descend. HR response is very different from EMG in the repeated stair ascend. Inactivity periods are clearly identifiable for analysis of duration and frequency. Note that ascend with concentric muscle work requires more EMG activity and energy than eccentric muscle action during walking down.

**Purpose**

This study aims to show in a cluster randomized controlled trial (RCT) that simple actions to reduce time in sitting position are viable and that they have health-related benefits. Parents and their young children are the primary target of the study. After tailored physical activity counseling the parents commit themselves to the agreed behavioral changes during work, leisure time and weekends. In leisure time and on weekends the change in behavior will be guided to activate also the children.

**The main specific purposes and hypotheses are:**

1. To test what is the effectiveness of tailored counseling to minimize inactivity periods and increase physical activity in persons involved in sitting-related work. *H: Counseling increases physical activity and decreases muscle inactivity time during workday and leisure time*
2. To test if the counseling can induce long term behavioral changes that benefit health?

*H: Counseling induces behavioral changes that last long time. Positive effects in health related markers such as serum lipids and blood pressure can be seen at 6 and 12 months after counseling.*

1. To examine what is the effectiveness of parental counseling on children’s physical activity behavior? *H: There is transfer effect of parental counseling so that also the children’s physical activity has increased.*

**Methods**

**Design.** A cluster randomized controlled intervention will be performed on parents that sit more than 50% of the work time. The intervention group will receive tailored counseling to decrease time in sitting position and increase daily activity, especially the non-exercise activity. Children aged 3-8 will be included to the study and their physical activity will be objectively monitored before and three times after the parental counseling.

**Subjects.** Eligible participants are parents with work that primarily includes sitting. *Inclusion criteria*: healthy men and women with children 3-8 years old, and having work where they sit more than 50% of their work time. *Exclusion criteria*: self-reported chronic, long-term musculoskeletal disease or progressive neurological disease, diagnosed cardiovascular or metabolic disease with regular medication, families with pregnant mother at baseline, BMI>35, children confined to a wheelchair or bed. The recruitment will be performed in Jyväskylä region delivering advertisements to parents via kindergartens and primary schools. To avoid contamination of treatment the randomization will be done regionally before recruitment and the control group will not be informed about the intervention. Both the intervention and control groups will include areas from the city center and suburbs of Jyväskylä.

**Sample size.** From the preliminary results it can be expected that small changes in behavior can effectively decrease the longest inactivity periods and total inactivity time as example in Fig.2 shows. Based on decrease in the inactivity periods by behavioral changes the sample size of 10 would be sufficient to show statistically significant changes. However, we also aim that the intervention has significant health effects. Thus, it was estimated that increase of 0.2 mmol/l in high-density lipoprotein after 6 or 12 months of intervention can be possible with the intervention. The required sample size to show this effect would be 77. Adding the cluster effect (Cambell et al. 2004) and drop-outs we estimate that 100 subjects in both groups would be needed.

Fig 2. Histogram of daily muscle activity time (% of whole day activity) divided in sections 0-5% of maximum (MVC). Women n=6, 30 measurement days, men n=6, 26 measurement days. The women performed more non-exercise activity than men and it is significantly reflected to the main outcome. **p<0,01, ***p<0,001 (Haakana 2010).

**Ethics.** We will submit the study plan with questionnaires and informed consent for evaluation by the local ethics committee. The RCT will not begin before approval has been received. Subjects will be volunteers with right to withdraw from the study at any time without consequences. The study will be conducted according to good scientific practice.

**Protocol.** The protocol of the study is shown in Fig. 3. Before counseling the parents will be measured for baseline data (fasting blood samples, anthropometry, questionnaires, physical fitness test) and a representative normal day EMG activity with other measures of physical activity level. After counseling the representative day with agreed behavioral changes will be measured again using EMG and other devices for physical activity level assessment. In the first 3 months the compliance will be reinforced by phone calls and possible modifications to the agreed intervention will be made. Between 3 to 6 months the compliance will be followed once a month and no contact with the subjects will be made in the maintenance period. The midline and endline measurements include fasting measurements, questionnaires and 24-hour heart rate recordings. Control group participates only in baseline and normal day measurements, midline and endline measurements. Children will be measured at baseline, after 3, 6, 9 and 12 months for physical activity behavior using triaxial accelerometers (Actigraf).

Figure 3. Timeline of the project showing the timing of physical activity (PA) measurements for parents and children.

**Measurements**


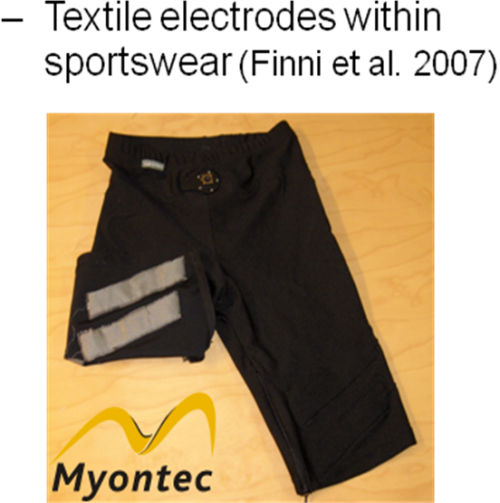
**Short term main outcome is the physical activity (PA) behavior**. In parents it will be assessed with EMG, triaxial accelerometer and questionnaires. ***EMG*** will be measured using shorts with embedded textile electrodes (Suunto Oy, Vantaa, Finland / Megaelectronics Ltd., Kuopio, Finland) measuring activity from 4 thigh muscles during normal daily life and laboratory tests (Fig. 4). The measurement system has been tested for validity, repeatability and feasibility in our laboratory (Finni et al. 2007). The data is collected to a 52 g module in the waist in raw form with sampling frequency of 1000Hz. The raw EMG signal is rectified and averaged over 100 ms non-overlapping intervals. The signals are stored in a module for off line-analysis. The EMG data will be analyzed for inactivity period times and frequencies as well as for total inactivity. This analysis will be repeated using different signal levels (1%, 2%, 5% of maximum voluntary contraction) for detection of the inactivity periods. Activity histograms and on-off analysis will be performed. Burst analysis (Kern et al. 2001) will be used to quantify activity periods.

In a sub group of children we test the use of EMG shorts as measurement tool of physical activity.

Fig. 4. Shorts that are used to collect muscle EMG activity from thigh muscles.

***Acceleration.*** Triaxial acceleration will be collected during all the measurements (Alive heart monitor, Alive technologies, Australia) and be used to compare the activity counts with EMG levels for validation purposes. **In children** the PA behavior will be measured using triaxial accelerometer (Actigraf) that the children wear on their waist for one week at pre, 3, 6, 9 and 12 months after counseling intervention to parents. During this one week period, the parents will fill in diary of the childrens physical activities. In addition, children’s basic motor skills will be tested three times during the intervention.

***Questionnaires.*** From the detailed 7-day physical activity questionnaire focusing on non-exercise activity the daily MET values will be calculated for the past year at baseline and endline (modified from Lakka et al. 1997). During the days of data collection, the subjects fill in detailed physical activity diaries that reveal standing up from sitting position, walked up stairs etc.

**Long term main outcomes are health-related indices and maintenance of the behavioral change.** Venous blood samples for whole blood count, blood lipids and glucose analysis will be taken in standardized fasting conditions in the mornings 7-9 am. Samples will be analyzed using standard methods in clinical use and also metabonomics. Serum will be stored for later analysis of other health related markers such as glucose and lipid metabolism (leptin, obestatin, glucagon-like peptide, C-peptide). DNA-samples will be taken from volunteers and leucocytes extracted for further analysis of associations regarding body composition and physical performance and activity. Blood pressure will also be measured in the morning. Anthropometry including body composition (dual X-ray absorbtiometry and bioimpedance, In-Body 720, Korea) in fasting condition, hip-to-waist circumference and BMI will be measured. Subcutaneous fat thicknesses from several sites will be measured using ultrasonography.

Heart rate will be measured with the same device as acceleration. Both time domain (e.g. RMSSD, SDNN) and frequency domain (Ultralow frequency <0.0033 Hz, Very Low frequency 0.0033-0.04 Hz, Low frequency 0.04-0.15 Hz and High frequency 0.15-0.4 Hz) analysis of HR variation (HRV) will be performed (Tulppo et al. 2003). Histogram of heart rate will be constructed. Hyvinvointianalyysi (Firstbeat Technologies, Finland) will be used to identify the time of physical activity, MET and stress-related decreases in HRV. The variables will be compared with EMG data and MET levels calculated from questionnaires. High frequency band has special meaning in determining cardiovascular health. Heart rate will be measured at baseline (before and after counceling), at 6 and 12 months.

Behavioral changes will be assessed using questionnaires that include questions such as: TV/computer time usage, time and places/facilities outdoors both alone and with family, way of commute, time in sitting position, subjective energy level etc.

**Background variables.** Background information of medication, chronic and acute diseases will be assessed in the screening process. Dietary records for 3 weekdays and 1 weekend day will be kept at baseline, midline and endline to evaluate whether similar diet has been maintained during the year as assumed. Questions regarding perceived health and wellbeing are included in the questionnaires. Physical fitness will be assessed with non-exercise questionnaire.

**Field measurements during normal day.** The subjects will be well instructed for the use of HR-acceleration monitor and EMG-garments. The data will be collected continuously for waking hours during normal daily life, so that the two days of data collection are similar in all other respects except that during the second day the agreed intervention program is executed. Both days, subjects are requested to do a simple reference test containing lying down, sitting, quiet standing and stationary squat that is used to control the reproducibility of the EMG signal during the day. Each morning the data collection will be started at the laboratory by research personnel, and ended by the subject before going to bed except for heart rate recordings that will continue overnight.

**Counseling intervention.** Tailored counseling by trained research assistants will be targeted to decrease time in sitting position and increase non-exercise activity during workdays (commute and work time) and leisure time (evenings and weekends). This type of personal counseling has been shown to be effective (Leinonen et al. 2007). First, the subjects will be asked for the use of bicycle, car, elevator, stairs and walks during the day to identify items that can be modulated, e.g. standing in coffee breaks, walking about while talking to phone, taking a walk before lunch etc. Leisure time habits will be asked and changes in habits are suggested such as walk to library instead of watching TV, going to visit neighbor by foot instead of calling on the phone. The items that the subjects are willing to modify will be written down and an agreement document will be signed by the subject and researcher to confirm the intended changes in behavior. Subjects will be motivated to this change by showing graphs such as Fig.1 and explaining the importance of muscle activity and pointing out long inactivity periods when one is sitting. Counseling includes encouragement toward family physical activities during leisure time (visiting playgrounds, swimming halls, parks and forests etc.) utilizing material from www.perheliikunta.net.

The counseling will take place about after 1 week of the baseline measurements. Starting from the parent’s baseline measurement the children will wear the accelerometers for 1 week. After this there will be the counseling and after 1 week from counseling the measurements for parents wearing EMG garments will be repeated on the same weekday as the baseline measurement.

After counseling the research personnel will phone the subjects two times to ask about the compliance of the goals set and send e-mails. This reinforcement period lasts 6 months. The end measurements at 12 months are made to assess the maintenance of the habitual changes and effects on health markers. During the last 6 months there will not be researcher contact other than 9 months measurements with the subjects.

The control group will not receive the counseling intervention but will undergo the measurements similarly as the intervention group. The counseling does not include dietary counseling. The subjects will be monitored for dietary changes by questionnaires but eating habits are assumed to be maintained the same.

**Statistical analysis**. The EMG activity variables such as total inactivity time during day and mean intensity of EMG will be used to categorize people into quartiles according to 1) physical activity and 2) health marker indexes. Chi Square will be used to compare EMG distributions and levels to other variables. The difference in physiological signals between categories will be compared using One-way/Multivariate ANOVA. Correlation and regression analysis will be used to retrieve relationships between physical and health conditions and physical activity assessments. Repeated measures ANOVA will be used to determine effects of intervention complemented with mixed model ANOVA in case of missing data points.

**Timetable**

Data collection for RCT baseline will begin in spring 2011 and continued through 2012 during spring and fall seasons. The seasonal variations in the subjects beginning the study in fall or spring will be different; however, during the baseline and endline measurements the weather allows similar outdoor activities.

### Budget

Funding for this project has been received from the Ministry of Education and Culture for 2011-2013.

### Expected research results and their practical usefulness

This research continues the PI’s project where the use of EMG to quantify physical activity level is unique. Our first results already demonstrate convincingly how little we use our muscles during normal daily life and that exercise has only limited effect on the muscle activity histograms (Finni et al. 2010ab, Haakana 2010).

As both our work and leisure time involve more sitting than ever before, the time and duration our muscles are active becomes attractive starting point for research because inactivity may induce negative effects on cellular processes in skeletal muscles or other tissues regulating risk factors like plasma triglycerides and HDL cholesterol (Hamilton et al 2007). Thus, while minimizing long periods of inactivity, we need to try to answer the question what is the frequency and duration of harmful inactivity, and vice versa, how much and how often do we need to activate our muscles or elevate HR.

The effectiveness of counseling intervention in minimizing the sitting time on muscle activity parameters will be realized in this randomized controlled study. We will also receive information if the intervention has reduced the need for occupational health care. If effective, the model of intervention can then be executed in occupational health care or through a company physician or team initiatives within work places, at homes and schools where the children benefit from this model, too. Indeed, now more than ever we must focus on children’s physical activity and inactivity. Based on social cognitive -theory (Bandura 2002) the amount of children’s physical activity and willingness to participate to different physical activities should increase if parents increase their own physical activity and take more time for physical activities with the whole family.

Therefore, the results of this study will result in information that has been especially called for in the ministry for 2007-2011: 1) Accurate and novel measure of physical activity using EMG to assess muscle activity and inactivity in addition to questionnaires, 2) Description and measure of non-exercise activity during the day in ordinary people, 3) How sitting and changes in sitting behavior can affect ordinary people’s health, fitness and non-exercise behavior and 4) Children’s physical activity behavior objectively measured by accelerometer.

**Publication plan**

This project will result in two Ph.D. theses which comprise of several original scientific articles. Most importantly, we aim that this study produces information that is relevant in various occupations from teachers to company executives. Thus, besides presenting the results at international scientific conferences and publishing in high-rank international peer-review journals we aim to write articles in the format understandable to public in various targeted magazines such as Liikunta ja Tiede, Opettaja, Taloussanomat etc. Also, the results regarding e.g. maximal inactivity periods will be published in newspapers and magazines to target the general population.

**References:**

ACSM’s guidelines for exercise testing and prescription. 2006. Lippincott Williams & Wilkins. 7^th^ ed.

Bandura, A. 2002. Sosiaalis-kongitiivinen teoria. Teoksessa R. Vasta (toim.) Kuusi teoriaa lapsen kehityksestä. Kuopio: UNIpress, 13-82.

Cambell et al. (2004) Consort statement: extension to cluster randomised trials. BMJ 328:702-708.

Finni, Hu, Kettunen, Vilavuo, Cheng. (2007) Measurement of EMG activity with textile electrodes embedded into clothing. Physiol Meas 28:1405-19.

Finni T, Haakana P, Tikkanen O, Petrin M, Pullinen T. (2010) Physical activity and inactivity druing normal daily life quantified using electromyography. 13^th^ World Sport for All Congress, Jyväskylä, Finland.

Finni T. (2010) Lihas lepää pääosan päivää – liikkuvallakin. Liikunta ja tiede, in press.

Gallahue, D. & Ozmun, J. 2006. Undertanding motor development. New York, NY.: McGraw Hill.

Haakana P. Assessment of physical activity in normal daily life using shorts with embedded EMG electrodes. Master’s thesis. University of Jyväskylä, 2010.

Hamilton, Hamilton, Zderic. Role of Low Energy Expenditure and Sitting in Obesity, Metabolic Syndrome, Type 2 Diabetes, and Cardiovascular Disease. Diabetes 56:2655–2667, 2007.

Healey et al. 2008. Breaks in sedentary time: beneficial associations with metabolic risk. Diabetes Care 31: 661-666.

Kern, Semmler, Enoka Long-term activity in upper- and lower-limb muscles of humans J Appl Physiol 2001: 91:2224-2232.

Lakka, Salonen. The physical activity questionnaires of the Kuopio Ischemic Heart Disease Study (KIDH). Med Sci Sports Exerc 29: S46-S58, 1997

Leinonen R et al. Customer-oriented counseling for physical activity in older people: study protocol and selected baseline results of a randomized-controlled trial (ISRCTN 07330512). Scand J Med Sci Sports. 2007 Apr;17(2):156-64.

Malina, R., Bouchard, C. & Bar-Or, O. 2004. Growth, maturation, and physical activity. Champaign, IL. : Human Kinetics.

Pellegrini, A. & Bjorklund, D. 1998. Applied child study. A developmental approach. Mahwah, NJ.: Lawrence Erlbaum Associates.

Rovio et al. 2009 Physically inactive adults in Finland – Identification of the subgroups. Liikunta & Tiede 46 (6), 26 – 33 & 13^th^ World Sport for all Congress, 14-17.6.2010 Jyväskylä.

Sjöström, Oja, Hagstömer, Smith, Bauman (2006) Health-enhancing physical activity across European Union countries: the Eurobarometer study. J Public Health 14:291-300.

Sääkslahti, A. 2005. Liikuntaintervention vaikutus 3-7-vuotiaiden lasten fyysiseen aktiivisuuteen ja motorisiin taitoihin sekä fyysisen aktiivisuuden yhteys sydän – ja verisuonitautien riskitekijöihin. Studies in Sport, Physical Education and Health 104.

Tulppo et al. Effects of aerobic training on heart rate dynamics in sedentary subjects. J Appl Physiol 95: 364–372, 2003.
